# Potassium Channel Conductance Is Involved in Phenylephrine-Induced Spontaneous Firing of Serotonergic Neurons in the Dorsal Raphe Nucleus

**DOI:** 10.3389/fncel.2022.891912

**Published:** 2022-06-06

**Authors:** Jing Wang, Yingzi Wang, Xiaona Du, Hailin Zhang

**Affiliations:** ^1^Department of Pharmacology, The Key Laboratory of Neural and Vascular Biology, Ministry of Education, The Key Laboratory of New Drug Pharmacology and Toxicology, Hebei Medical University, Shijiazhuang, China; ^2^Department of Pharmacochemistry, Hebei University of Chinese Medicine, Shijiazhuang, China

**Keywords:** serotonergic neuron, phenylephrine, dorsal raphe nucleus, activity, A-type K^+^ channels, Kv7/KCNQ K^+^ channels, calcium-activated small-conductance K^+^ (SK) channels

## Abstract

The serotonergic (5-HT) network from the dorsal raphe nucleus (DRN) of the brain has been demonstrated to regulate cognition, emotion, and behaviors, including learning and the sleep-wake cycle. Dysregulation of the activity of 5-HT neurons in the DRN is thought to play an important role in emotional disorders. The activity of 5-HT neurons is regulated by norepinephrine (NE) released from the projection terminals of noradrenergic input from the locus coeruleus (LC) *via* activation of the α1-adrenoceptor. However, insight into the molecular mechanism underlying this NE-induced regulation of 5-HT neuron activity is not clear. In this study, using the agonist of α1-adrenoceptor phenylephrine (PE), brain slices, and patch clamp, we found that A-type, Kv7/KCNQ, and calcium-activated low-conductance K^+^ channels (SK) underlie PE-induced spontaneous firing in DRN 5-HT neurons. Using single-cell PCR and immunofluorescence, we also identified the isoforms of these K^+^ channel families that might contribute to the NE/PE-induced spontaneous firing of DRN 5-HT neurons.

## Introduction

The serotonergic (5-HT) system originating in the dorsal raphe nucleus (DRN) plays a central role in multiple important brain functions, including learning, cognition, emotion, and the sleep-wake cycle (Lucki, 1998; Monti, 2010; Kawashima, 2018). There is ample evidence that the activity of DRN 5-HT neurons is correlated with reward levels (Nakamura et al., 2008; Bromberg-Martin et al., 2010; Hayashi et al., 2015), aversive stimuli (Schweimer and Ungless, 2010; Hayashi et al., 2015), and the absence of rewards (Li et al., 2013). Moreover, altered activity of these DRN 5-HT neurons is associated with the response to stress and the onset of psychiatric disorders such as major depressive disorder (MDD) and anxiety (Ohmura et al., 2020; Prakash et al., 2020; Zou et al., 2020). For a better understanding of the activity-dependent role of DRN 5-HT neurons in the above physiological and pathological processes, it is essential to understand the molecular/ionic mechanisms underlying the electrical discharge activity of these neurons.

In most species, the 5-HT neurons of the DRN fire electrical discharges when recorded *in vivo*, with a slow, tonic firing pattern at typical frequency of about 0.5–3 Hz (Aghajanian et al., 1968; Aghajanian and Vandermaelen, 1982; Allers and Sharp, 2003). However, when recorded *in vitro* in brain slices, the 5-HT neurons are silent and do not fire spontaneously unless triggered by norepinephrine (NE), the transmitter released in the DRN mainly from the projection terminals of the noradrenergic input from the locus coeruleus (LC). In this case, the 5-HT neurons fire spontaneously after either NE or the α_1_-adrenoceptor agonist phenylephrine (PE) is applied to the brain slices (Vandermaelen and Aghajanian, 1983; Pan et al., 1990; Judge and Gartside, 2006). These results suggest that noradrenergic modulation is critical for the firing activity of DRN 5-HT neurons. However, the molecules/ion channels responsible for this NE-induced modulation have not been elucidated.

Early studies suggest that NE or PE-induced firing initiation of DRN 5-HT neurons appears to depend in large part on the closure of membrane K^+^ conductance (Aghajanian, 1985; Leonard, 2002), with the involvement of A-type K^+^ currents (I_A_) (Aghajanian, 1985). However, this mechanism has not been clearly elucidated. Subthreshold K^+^ conductance is the most important determinant for triggering neuron firing. An example of this is the Kv7/KCNQ/M current (I_M_). KCNQ/M-type currents have been shown to be involved in the regulation of spontaneous firing of central neurons, including DRN 5-HT neurons (Zhao et al., 2017; Su et al., 2019). In addition, KCNQ channels in chemosensitive neurons of the retrotrapezoid nucleus (RTN) have been reported to be the downstream effectors of NE modulation of RTN activity (Kuo et al., 2016). However, it is not known if KCNQ/M-type currents are also involved in NE-induced spontaneous firing of DRN 5-HT neurons. Another potential candidate for the K^+^ conductance relevant here is the calcium-activated low-conductance K^+^ channel (SK channel). Several conflicting reports on the effect of NE on SK channels through activation of the α_1_-adrenoceptor have been published for neurons in both central and peripheral nervous systems, including the DRN 5-HT neurons (Pan et al., 1994; Wagner et al., 2001; Maingret et al., 2008). However, there is no direct evidence for NE inhibition of SK channel currents in DRN 5-HT neurons.

In this study, we sought to find the K^+^ channels underlying the NE/PE-induced spontaneous firing of DRN 5-HT neurons, focusing on the A-type, KCNQ/M, and SK K^+^ channels. Using brain slice preparations and patch clamp, we demonstrate that PE triggers the activity of DRN 5-HT neurons through the α_1_-adrenoceptor and inhibition of the A-type, KCNQ/M, and SK K^+^ channels. Using single-cell PCR and immunofluorescence, we also identified the isoforms of these K^+^ channel families that might contribute to the NE/PE-induced spontaneous firing of DRN 5-HT neurons.

## Materials and Methods

### Animal Preparation

Male 6- to 8-week-old C57BL/6 mice (Vital River, China) were used for the studies. All experiments were performed in accordance with the guidelines of the Animal Care and Use Committee of Hebei Medical University and approved by the Animal Ethics Committee of Hebei Medical University.

### Ethics Statement

All experiments were performed in accordance with the guidelines of Animal Care and Use Committee of Hebei Medical University.

### Brain Slice Preparation

The details for preparation of coronal brain sections containing DRN were the same as described in our previous work (Zhao et al., 2017). Briefly, mice were anesthetized with chloral hydrate [200 mg/kg, intraperitoneally (i.p.)]. After intracardial perfusion with an ice-cold sucrose solution (260 mM sucrose, 25 mM NaHCO_3_, 2.5 mM KCl, 1.25 mM NaH_2_PO_4_, 2 mM CaCl_2_, 2 mM MgCl_2_, and 10 mM D-glucose; osmolarity, 295–305 mOsm; saturated with 95% O_2_ and 5% CO_2_), the brains of the mice were removed quickly and placed into the slicing solution. Coronal midbrain slices (200 μm thick) containing DRN (AP −3.8 to −4.8 mm; LM 0 mm; and DV −2.8 to −3.8 mm) were sectioned with a vibratome (VT1200S; Leica, Germany). The sections were incubated for 30 min at 36°C in oxygenated artificial cerebrospinal fluid (ACSF) (in mM: 124 NaCl, 3 KCl, 1.25 NaH_2_PO_4_, 2 CaCl_2_, 2 MgCl_2_, 25 NaHCO_3_, 10 D-glucose; osmolarity, 280–300 mOsm), and stored at room temperature for 90 min (23–25°C) before use.

### Identification of 5-HT Neurons and Electrophysiological Recordings

5-HT neurons located in the midline of the ventromedial subdivisions of the DRN were used. DRN 5-HT neurons were identified by single-cell PCR for the presence of tryptophan hydroxylase (TPH). Recordings in the slices were performed in whole-cell voltage-clamp configurations on a Multiclamp 700B amplifier coupled with a Digidata 1440A AD converter (Molecular Devices, United States) using borosilicate patch electrodes (1–3 MΩ) wrapped with parafilm to reduce pipette capacitance. Pipette series resistance (typically 4–8 MΩ) was compensated by 70–85% during voltage-clamp experiments and was checked frequently throughout the experiment; data were not used if series resistance changed by >15%. Voltage signals were filtered at 10 kHz and sampled at 20 μs using a Digidata 1440A data acquisition interface (Molecular Devices) and pClamp 9 software (Molecular Devices). For recording K^+^ currents, glass electrodes (3–5 MΩ) were filled with the following internal solutions, namely, 115 mM K-methylsulfate, 20 mM KCl, 1 mM MgCl_2_, 10 mM *N*-2-hydroxyethylpiperazine-*N*′-2-ethanesulfonic acid (HEPES), 0.1 mM EGTA, 2 mM MgATP, and 0.3 mM Na_2_GTP, pH adjusted to 7.4 with KOH. For recording I_M_ and SK currents, ACSF was used as extracellular solution. For recording I_A_ current, HEPES-buffered ACSF (130 mM NaCl, 4 mM KCl, 2 mM CaCl_2_, 2 mM MgCl_2_, 10 mM HEPES, 10 mM D-glucose; 280–300 mOsm) was used as extracellular solution. To optimally isolate the outward SK currents from other K^+^ currents and the Na^+^ currents, 5 mM tetraethylammonium (TEA) and 1 μM tetrodotoxin were added to the extracellular solution in voltage-clamp experiments (Sailer et al., 2002; Pedarzani et al., 2005). For isolating A-type currents (I_A_), 1 μM tetrodotoxin was used to block fast voltage-activated Na^+^ channels, and 0.3 μM CdCl_2_ was used to block voltage-activated Ca^2+^ channels (Itri et al., 2010; Hu et al., 2019).

For recording spontaneous firing of the neurons, cell-attached “loose-patch” (100–300 MΩ) recordings were used (Burlet et al., 2002). In this case, patch pipettes (2–4 MΩ) were filled with ACSF, and the spontaneous activity was recorded in the current-clamp mode (I = 0). All of the experiments were performed at room temperature (25 ± 2°C). In our recordings, the majority of the recorded neurons, ∼90%, were silent without added PE, and indeed a small number of recorded cells (∼10%, 10 out of 100) had activity of spontaneous firing. However, these neurons with spontaneous firing were found mostly to be either dopaminergic neurons or glutamatergic neurons as verified by the single-cell PCR post the electrophysiological recordings (*n* = 8, 80% dopaminergic neurons; *n* = 1, 10% glutamatergic neurons). The dopamine and glutamatergic neurons in DRN were also reported in the literature (Soiza-Reilly and Commons, 2011; Matthews et al., 2016; Commons, 2020). Therefore, neurons exhibiting spontaneous activity were not included for data analysis.

### Immunofluorescence

After intracardial perfusion with 4% paraformaldehyde (PFA) in 0.01 M phosphate-buffered saline (PBS) (pH 7.4), followed by 0.01 M PBS, mice brains were post-fixed in 4% PFA at 4°C for 48 h and coronal midbrain slices were prepared. Sections (50 μm thick) were blocked with 0.3% Triton X-100 and 10% donkey serum (Biological Industries, Israel) in PBS and incubated overnight at 4°C with a mixture of primary antibodies. Sections were then washed with PBS three times (5 min) and then incubated in a mixture of secondary antibodies at room temperature for 2 h. Sections were then washed three times (7 min) with PBS. Finally, slices were mounted with Prolong Gold antifade reagent (Life Technologies, United States). Images were obtained on a Leica TCS SP5 confocal laser microscope (Leica, Germany) equipped with laser lines for FITC (Argon 488) and cy3 (HeNe 543). Images were analyzed with LAS-AF-Lite software (Leica, Germany).

### Single-Cell PCR

PrimeScript™ II 1st Strand cDNA Synthesis Kit (Takara-Clontech, Japan) was used to perform reverse transcription. At the end of electrophysiological recordings, the recorded cell was aspirated into a pipette and then expelled into a sterile PCR tube containing 1 μl oligo-dT primer and 1 μl dNTP mixture. The mixture was heated to 65°C for 5 min and then cooled on ice for 2 min. Synthesis of the first single-strand cDNA from the cellular mRNA was performed with PrimeScript II reverse transcriptase (Takara) at 50°C for 50 min and then 85°C for 5 min. cDNA was stored at −20°C. Then, single-strand cDNA was amplified using GoTaq Green Master Mix (Promega, United States). Two rounds of conventional PCR with pairs of gene-specific outer (first round) and inner primers (second round) for GAPDH (positive control), TPH, Kv4.1–4.3, 3.3, 3.4, Kv1.4, SK1–3, and KCNQ1–5 were performed. After adding the specific outer primer pairs into each PCR tube (final volume 25 μl), first-round synthesis was performed as follows, namely, 95°C (5 min); 30 cycles of 95°C (50 s), 60°C (50 s), 72°C (50 s); 72°C (5 min). Then, 2 μl of the first PCR product were used for the second amplification with specific inner primers (final volume 25 μl). The second-round amplification was performed as follows, namely, 95°C (5 min); 35 cycles of 95°C (50 s), 58–63°C (45 s), 72°C (50 s) and 5 min elongation at 72°C. The final PCR products were separated by electrophoresis on 2% agarose gels. Negative control reactions with no added template were included in each experiment.

The “outer” primers (from 5′ to 3′) were as follows:

**Table:** 

GAPDH	AAATGGTGAAGGTCGGTGTGAACG (sense)	AGTGATGGCATGGACTGTGGTCAT (antisense)
TPH	GAGTCCTCATGTACGGCACC	AGGCCGAACTCGATTGTGAA
Kv1.4	CTCTGGGCTCCACTAACGAG	CTTCTCAGAGACTCGGCGTT
Kv3.3	TGCTCAACTACTACCGCACC	AAGAATAGGGAGGCGAAGGC
Kv3.4	ACGTGACGGAGATTCATCGG	TCTTGAAGTCGGTGTGGTCG
Kv4.1	ACCACACTTGGGTATGGAG	TGAACTCGTGACACGTAGTCTTCT
Kv4.2	CGCTCTGATAGTGCTGAACG	CCTGCGGTCCTTGTACTCCT
Kv4.3	ATGCATCTCTGCCTACGACG	CTGCGGATGAAGCGGTATCT
KCNQ1	CCCAGTGCTGAAAGGAAGCG	ACGAAACACTTCCAACCCGT
KCNQ2	TCATCCCACCTCTGAACCAG	TGGGCGCAGACTCTCTTTG
KCNQ3	AGACGTGGAGCAAGTCACCTT	CCAGCCTTTGTATCGACAGC
KCNQ4	CCCGGGTGGACCAAATTGT	AGCCCTTCAGTCCATGTTGG
KCNQ5	GAAGCCGCTCTCCTACACC	TTCTGTCCATGCGCACCATA
SK1	GTCTCCTCCTGGATCGTTGC	CTTGGTGAGCTGTGTGTCCAT
SK2	ACCCTAGTGGATCTGGCAAAG	GAGCGCTCAGCATTGTAGGA
SK3	GGCGGATAGCCATGACCTAC	AAAGGTCCACCAGGGTGTTG

The “inner” primers (from 5′ to 3′) were as follows:

**Table:** 

GAPDH	GCAAATTCAACGGCACAGTCAAGG	TCTCGTGGTTCACACCCATCACAA
TPH	TGGCTACAGGGAAGACAACG	GTATCTGGTTCCGGGGTGTA
Kv1.4	GACAACCGAACTTGTTCCGT	GTCTTAGCACTTGCCTTCTC
Kv3.3	GGGCTTCTGGGGCATAGAC	GTCCTGAAAACACAGACGCTT
Kv3.4	TTGTGTGCTGCCCTGATACG	GACAAACCACTCAATCCCACC
Kv4.1	TTGGGTCCATCTGCTCACTT	GGCCCCCATTTTGCTTATAC
Kv4.2	CCTGGAACGATACCCAGACAC	CCCGTGCGGTAGAAGTTGA
Kv4.3	AGCTTCCGTCAGACCATGTG	GGCAAAAGAAAGCCACCGAAT
KCNQ1	GTGTCCCTTCTCACTGGAGC	CACTGTAGATGGAGACCCGC
KCNQ2	CATCACCAAGTCAGAAGGTCAG	ACAAACTCGCAGTTACAGCTC
KCNQ3	CAAGTACAGGCGCATCCAAAC	GGCCAGAATCAAGCATCCCA
KCNQ4	ATGGGGCGCGTAGTCAAGGT	GGGCTGTGGTAGTCCGAGGTG
KCNQ5	GTTCGTCTACCACGCGTTC	CGAGCAAACCTCAGTCTTCC
SK1	ATGGTGCCGCATACCTACTG	CACGTGTTTCTCAGCCTTGG
SK2	GGATCTGGCAAAGACCCAGAAT	AGGGAGGGCATGAATGCTAC
SK3	CCCCATCCCTGGAGAGTACA	TTCACAGACTCGCACAGTCC

### Drugs

All drugs were bath applied at the following concentrations, namely, PE (10 μM; Tocris), prazosin (5 μM; Sigma), apamin (500 nM; Sigma), 4-aminopyridine (4-AP; 4 mM; Sigma), XE991 (3 μM; Tocris), 6-cyano-7-nitroquinoxaline-2,3-dione (CNQX; 10 μM; Sigma), DL-2-amino-5-phosphonopentanoic acid (APV; 50 μM; Sigma), strychnine (2 μM; Sigma), and gabazine (10 μM; Sigma).

Commercial antibodies used were anti-TPH (1:400, mouse, sigma, T0678, RRID:AB_261587), anti-Kv4.2 (1:400, rabbit, AlomoneLabs, APC-023, RRID:AB_2040176), anti-Kv4.3 (1:400, rabbit, AlomoneLabs, APC-017, RRID:AB_2040178), anti-SK2 (1:200, rabbit, Bioss, DF13499, RRID:AB_2846518), anti-SK3 (1:200, rabbit, Proteintech, 17188-1-AP), anti-KCNQ2 (1:200, goat, Santa Cruz, sc-7793, RRID:AB_2296585), anti-KCNQ3 (1:200, goat, Santa Cruz, sc-7794, RRID:AB_2131714), and anti-KCNQ4 (1:200, rabbit, AlomoneLabs, APC-164, RRID:AB_2341042). Secondary antibodies used were donkey anti-mouse IgG (H + L) highly cross-adsorbed secondary antibody (Alexa Fluor 568, Thermo Fisher Scientific, A10037, RRID:AB_2534013, 1:1,000), donkey anti-rabbit IgG (H + L) highly cross-adsorbed secondary antibody (Alexa Fluor 488, Thermo Fisher Scientific, A-21206, RRID:AB_2535792, 1:1,000), and donkey anti-goat IgG (H + L) cross-adsorbed secondary antibody (Alexa Fluor 488, Thermo Fisher Scientific, A-11055, RRID:AB_2534102, 1:1,000).

### Statistics

All data are expressed as mean ± SEM. Group size (*n*) indicates the number of independent, non-technical replicates. For electrophysiological data, the discharge rate and the current amplitudes were compared using the paired *t*-test, when data were normally distributed and there was no significant variance inhomogeneity. When normality or equal variance of samples was not present, the Wilcoxon matched-pairs signed-rank test was used. *p*-Values ≤ 0.05 were accepted as statistically significant. Data analysis was carried out using GraphPad Prism 6.0 (RRID:SCR_002798).

## Results

**FIGURE 1:**
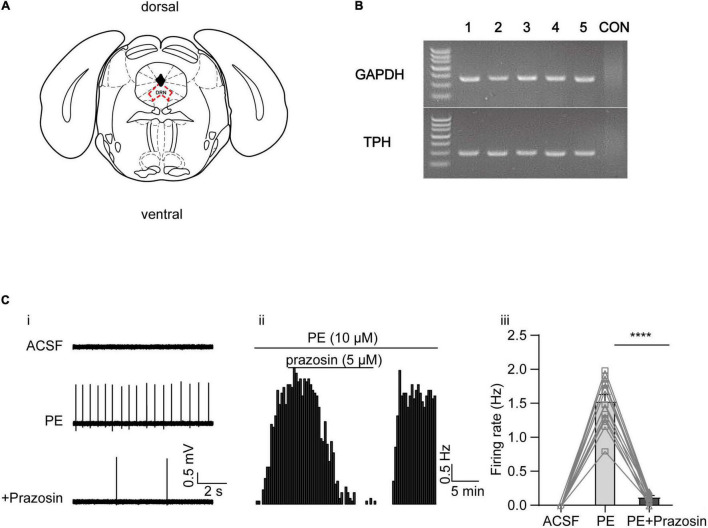
Phenylephrine induces spontaneous firing in the DRN 5-HT neurons by activating α1 adrenergic receptor. **(A)** Schematic illustration of the dorsal raphe nucleus (DRN) on a coronal plane. **(B)** Representative image of single-cell PCR products displayed on a gel, from five DRN neurons (lanes 1–5), and a negative control (“CON”) in which no DNA template from neurons was added. TPH, tryptophan hydroxylase, marker of 5-HT neurons. **(C)** PE induced the spontaneous firing activity in the DRN 5-HT neurons, which was inhibited by α1 adrenergic receptor blocker prazosin. **(i)** Representative traces of action potential spikes recorded using “loose patch” under current-clamp configuration performed on a DRN 5-HT neuron; the effects of PE (10 μM) and prazosin (5 μM) are shown. **(ii)** Example time course of the spontaneous firing frequency in a 5-HT neuron, under the influence of PE and prazosin. **(iii)** Summarized effects of PE and prazosin on the spontaneous firing frequency (paired *t*-test, ****p < 0.0001, *n* = 13).

Previous studies have shown that activation of the α_1_-adrenoceptor (by NE or PE) in DRN 5-HT neurons is associated with a depolarization of resting membrane potential and an increase in input resistance, likely due to reduced K^+^ conductance (Aghajanian, 1985). However, the identity of this K^+^ conductance is not known. After reviewing the experimental evidence in the literature described in the introduction, we aimed to study three families of K^+^ channels, namely, the A-type, the KCNQ/M, and the SK channels. We first verified that PE, the selective α1-adrenoceptor agonist, elicits spontaneous firing of DRN 5-HT neurons that were otherwise silent. Firings of the neurons were recorded in DRN brain slices using a “loose cell-attached patch” method (Burlet et al., 2002), and the recorded neurons were located in the midline in the ventromedial subdivision of the DRN (Figure 1A), as 5-HT neurons are reported to be most densely located in this region (Gocho et al., 2013). 5-HT neurons were identified by the presence of TPH in single-cell PCR analysis after electrophysiological recordings (Figure 1B). PE (10 μM) significantly induced a slow (<5 Hz), clock-like discharge of action potentials in DRN 5-HT neurons, which was inhibited by the α_1_-adrenoceptor antagonist prazosin (5 μM) (Figure 1C).

### Role of the A-Type K^+^ Current in Phenylephrine-Induced Spontaneous Firing of Dorsal Raphe Nucleus 5-HT Neurons

**FIGURE 2:**
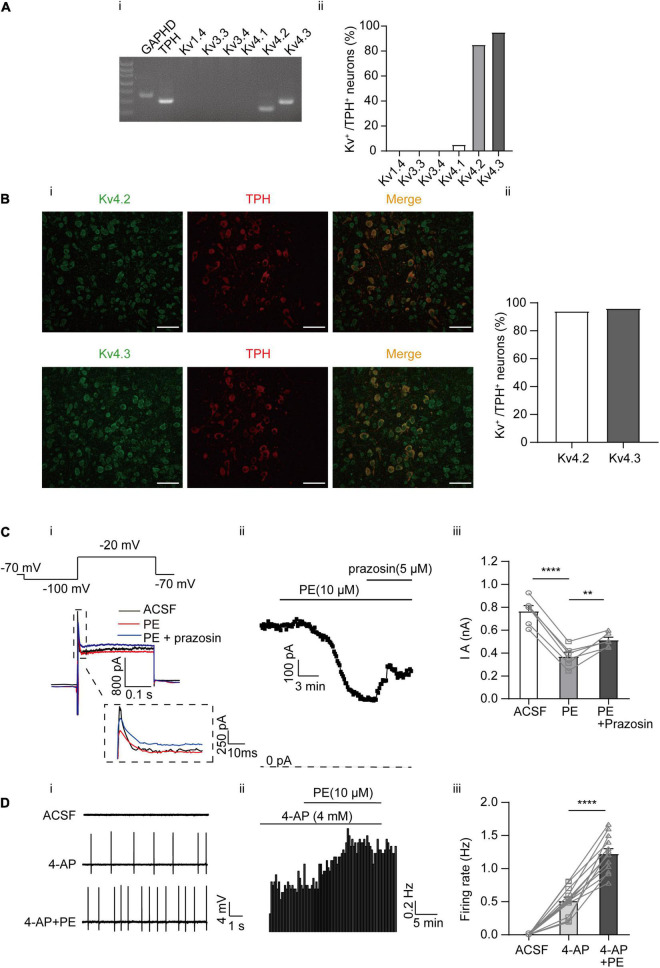
Expression of A-type K^+^ channels and their contribution to the PE-induced spontaneous firing of the DRN 5-HT neurons. **(A)** Expression of A-type K^+^ channel-related subfamily members assessed using single-cell PCR analysis in the DRN neurons. **(i)** Representative image of single-cell PCR products showing the presence of different Kv subunits. **(ii)** Proportion of Kv1.4, 3.3, 3.4, and Kv4s-positive neurons in the TPH-positive neurons (*n* = 20). **(B) (i)** Confocal images of Kv4.2 and 4.3 protein expression in slice of the DRN, assessed using immunofluorescence methods. Scale bar = 50 μm. **(ii)** Proportion of Kv4.2 and 4.3-positive neurons in the TPH-positive neurons (*n* = 200). **(C)** PE potently inhibited A-type currents recorded using whole-cell patch clamp in the DRN 5-HT neurons. **(i)** Recording protocol used and the typical current traces recorded; the latter were from –20 mV. The current amplitude at the beginning of the –20 mV step was measured (dotted square, enlarged in inset). **(ii)** Time course for current amplitudes measured in **(i)**. **(iii)** Summarized data for experiments shown in **(i,ii)**. Paired *t*-test, **p < 0.01, ****p < 0.0001, *n* = 6. **(D)** A-type K^+^ channel blocker 4-AP (4 mM) induced spontaneous firing of the DRN 5-HT neurons. **(i)** Representative traces of action potential spikes recorded using “loose patch” under current-clamp configuration. **(ii)** Example time course of the spontaneous firing frequency in a 5-HT neuron, under the influence of 4-AP and PE. **(iii)** Summarized data for the effects of 4-AP and PE on the spontaneous firing frequency (paired *t*-test, ****p < 0.0001, *n* = 13).

In a previous study, A-type currents (I_A_) were found to be inhibited by PE in DRN 5-HT neurons. However, it was not tested whether this inhibition contributes to the PE-induced firing of these neurons (Aghajanian, 1985). Moreover, the expression profiles of A-type channel in DRN 5-HT neurons have not been investigated. Therefore, we first examined the subtypes of A-type K^+^ channels expressed in DRN 5-HT neurons using single-cell PCR and immunofluorescence analysis. Single-cell PCR results revealed a strong expression of Kv4.2 and 4.3, a weak expression of Kv4.1, and no detectable expression of Kv1.4, Kv3.3, and Kv3.4 (Figures 2Ai,ii). Expression of Kv4.2 and Kv4.3 proteins in DRN 5-HT neurons was also confirmed by immunofluorescence, which showed strong signals for these channel proteins (Figure 2B), consistent with the results of single-cell PCR. These results suggest that Kv4.2 and 4.3 are the dominant A-type K^+^ channels in DRN 5-HT neurons and mediate the majority of I_A_. Next, we investigated the role of these A-type K^+^ channels in PE-induced firing of 5-HT neurons. Synaptic blockers (CNQX, APV, and gabazine) were added to isolate the intrinsic firing properties and I_A_ were recorded using the protocol shown in Figure 2Ci; the cells were voltage-clamped at −70 mV, followed by a hyperpolarizing step to −100 mV (200 ms), and then a step depolarization to −20 mV (300 ms). I_A_ were isolated as characteristic transient currents with rapid activation and inactivation, measured as instantaneous currents at the beginning of the −20 mV step. Bath application of PE (10 μM) significantly reduced peak I_A_ currents (from 0.77 ± 0.05 to 0.37 ± 0.04 nA, *n* = 6, *p* < 0.0001, paired *t*-test), and this reduction was significantly reversed by prazosin (5 μM; Figure 2C), the antagonist of α_1_-adrenoceptors. This result, in combination with the results shown in Figure 1, suggests that inhibition of I_A_ currents contributes to the PE-induced spontaneous firing of DRN 5-HT neurons. To confirm this, we tested the effect of A-type channel blocker, 4-AP. 4-AP at maximal I_A_ inhibiting concentration (4 mM) (Yao and Tseng, 1994; Serôdio et al., 1996; Song et al., 1998) also induced spontaneous firing in DRN 5-HT neurons, suggesting that inhibition of I_A_ indeed triggers spontaneous firing. However, in the continued presence of 4-AP, PE (10 μM) further increased the firing frequency from 0.51 ± 0.06 to 1.23 ± 0.07 Hz (*n* = 13, *p* < 0.0001, paired *t*-test, Figure 2D) in a statistically significant manner, indicating that another mechanism besides I_A_ inhibition was involved in the PE-induced spontaneous firing of DRN 5-HT neurons.

### Role of KCNQ/M-Type Current in Phenylephrine-Induced Spontaneous Firing of Dorsal Raphe Nucleus 5-HT Neurons

**FIGURE 3:**
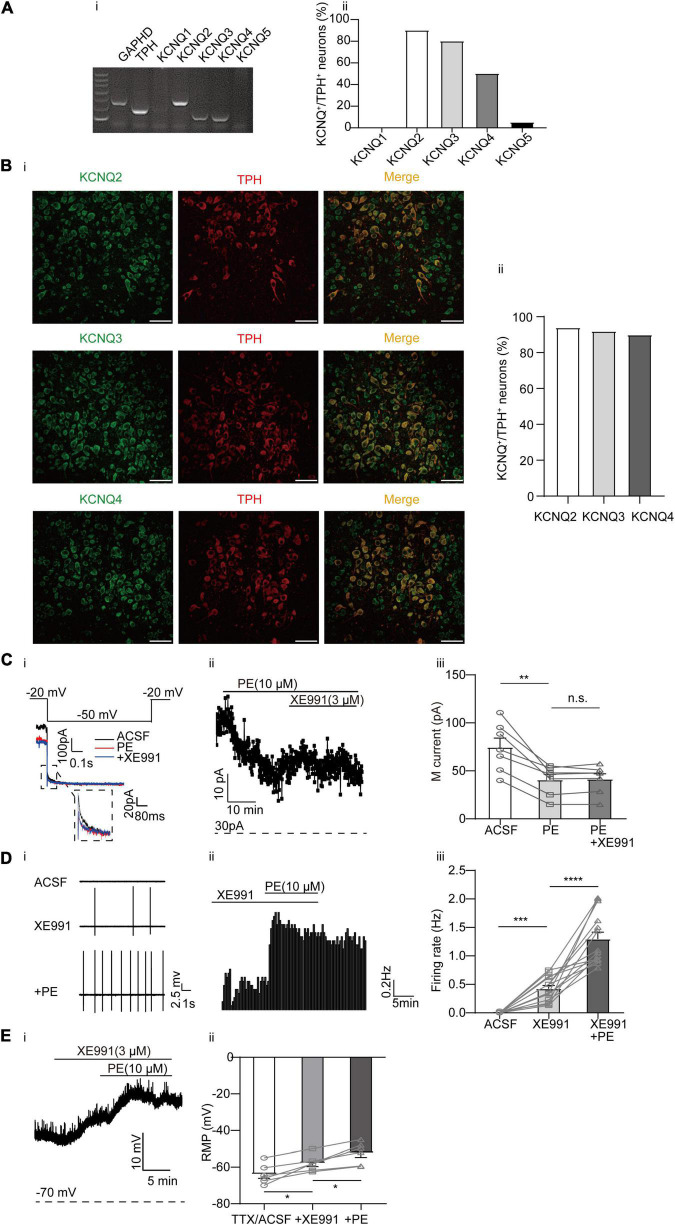
Expression of M-type K^+^ channels and their contribution to the PE-induced spontaneous firing of the DRN 5-HT neurons. **(A)** Expression of M-type K^+^ channel-related subfamily members assessed using single-cell PCR analysis in the DRN neurons. **(i)** Representative image of single-cell PCR products showing the presence of different KCNQ subunits. **(ii)** Proportion of KCNQ1–5 positive neurons in the TPH-positive neurons (*n* = 20). **(B) (i)** Confocal images of KCNQ2, KCNQ3, and KCNQ4 expression in slice of the DRN, assessed using immunofluorescence methods. Scale bar = 50 μm. **(ii)** Proportion of KCNQ2, KCNQ3, and KCNQ4 positive neurons in the TPH-positive neurons (*n* = 200). **(C)** PE potently inhibited M-type currents recorded using whole-cell patch clamp in the DRN 5-HT neurons. **(i)** Recording protocol used and the typical current traces recorded; the latter were from –20 mV. The current amplitude at the beginning of the –50 mV step was measured (dotted square, enlarged in inset). The effects of PE and M-type K^+^ channel blocker (XE991, 3 μM) are shown. **(ii)** Time course for current amplitudes measured in **(i)**. **(iii)** Summarized data for experiments shown in **(i,ii)**. Paired *t*-test, *^n.s.^p* > 0.05, **p < 0.01, *n* = 7. **(D)** M-type K^+^ channel blocker XE991 (3 μM) induced spontaneous firing of the DRN 5-HT neurons. **(i)** Representative traces of action potential spikes recorded using “loose patch” under current-clamp configuration. **(ii)** Example time course of the spontaneous firing frequency in a 5-HT neuron, under the influence of 4-AP and PE. **(iii)** Summarized data for the effects of XE991 and PE on the spontaneous firing frequency (Wilcoxon matched-pairs signed-rank test, ***p < 0.001, ****p < 0.0001, *n* = 14). **(E)** M-type K^+^ channel blocker XE991 (3 μM) induced depolarization of resting membrane potential of the DRN 5-HT neurons. **(i)** Example time course of the resting membrane potential in a 5-HT neuron, under the influence of 4-AP and PE. **(ii)** Summarized data for the effects of XE991 and PE on the resting membrane potential (Paired *t*-test, *p < 0.05, *n* = 6).

Our previous studies have shown that the KCNQ4 channel is the predominant Kv7/KCNQ isoform expressed in DRN 5-HT neurons (Zhao et al., 2017), although other neuronal KCNQ members (KCNQ2, KCNQ3, and KCNQ5) in these neurons have not been studied. In this study, the results of single-cell PCR analysis revealed robust expression of KCNQ2, KCNQ3, and KCNQ4 mRNA in DRN 5-HT neurons (Figures 3Ai,ii). Consistently, the result of immunofluorescence analysis showed high expression levels of the KCNQ2, KCNQ3, and KCNQ4 proteins in DRN 5-HT neurons (Figure 3B). To correlate the PE-induced spontaneous firing with its modulation of KCNQ/M-type currents, we first examined whether PE could inhibit KCNQ/M-type currents in DRN 5-HT neurons. M-type currents were measured using the protocol shown in Figure 3Ci as characteristic slow deactivating tail currents at a −50 mV step from a depolarized potential of −20 mV (Zhao et al., 2017). As shown in Figure 3C, PE (10 μM) significantly inhibited the M-type currents from 74.67 ± 9.46 to 40.63 ± 5.69 pA (*n* = 7, *p* < 0.01, paired *t*-test). Subsequently, it was found that XE991, a selective KCNQ blocker, failed to further inhibit the M-type currents, indicating complete inhibition of this K^+^ conductance by PE. Moreover, inhibition of M-type currents by XE991 resulted in depolarization of the resting membrane potential (from −63.91 ± 2.21 to −57.87 ± 1.83 mV, *n* = 6, *p* < 0.05, paired *t*-test, Figure 3E). With continued presence of XE991, PE (10 μM) further depolarized in a significant manner the resting membrane potential to −52.27 ± 2.47 mV. These results suggest that PE-induced inhibition of M-type current might trigger the neuronal firing. Next, we showed that blocking M-type current by addition of XE991 (3 μM) produced spontaneous firing of DRN 5-HT neurons (0.42 ± 0.06 Hz, *n* = 14, *p* < 0.001, Wilcoxon matched-pairs signed-rank test, Figure 3D). These results indicate that inhibition of KCNQ/M-type currents contributes to the PE-induced spontaneous firing of the DRN 5-HT neurons. Consistent with the involvement of multiple K^+^ channels in the PE-induced spontaneous firing of the DRN 5-HT neurons, the XE991-induced firing rate was further increased when PE was applied (1.30 ± 0.12 Hz, *n* = 14, *p* < 0.0001, Wilcoxon matched-pairs signed-rank test, Figure 3D).

### Role of Low-Conductance Ca^2+^-Activated K^+^ Current in Phenylephrine-Induced Spontaneous Firing of Dorsal Raphe Nucleus 5-HT Neurons

**FIGURE 4:**
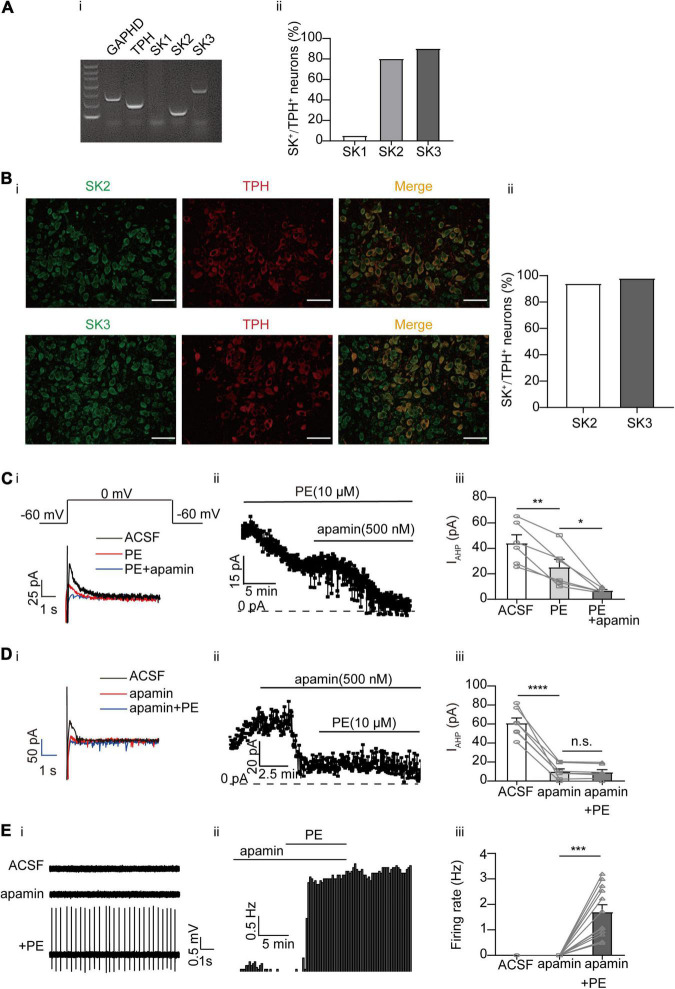
Expression of SK channels and their contribution to the PE-induced spontaneous firing of the DRN 5-HT neurons. **(A)** Expression of SK channel-related subfamily members assessed using single-cell PCR analysis in the DRN neurons. **(i)** Representative image of single-cell PCR products showing the presence of different SK subunits. **(ii)** Proportion of SK1–SK3-positive neurons in the TPH-positive neurons (*n* = 20). **(B) (i)** Confocal images of SK2 and SK3 expression in slice of the DRN, assessed using immunofluorescence methods. Scale bar = 50 μm. **(ii)** Proportion of SK2 and SK3 positive neurons in the TPH-positive neurons (*n* = 200). **(C)** PE potently inhibited I_*AHP*_ currents, which was further inhibited by SK channel blocker apamin, recorded using whole-cell patch clamp in the DRN 5-HT neurons. **(i)** Recording protocol used and the typical current traces recorded; the latter were from 0 mV. The current amplitude at the beginning of the 0 mV step was measured; the effects of PE (10 μM) and apamin (500 nM) are shown. **(ii)** Time course for current amplitudes measured in **(i)**. **(iii)** Summarized data for experiments shown in **(i,ii)** (paired *t*-test, **p < 0.01, *p < 0.05, *n* = 6). **(D)** PE did not further inhibit I_*AHP*_ current in DRN 5-HT neurons, following the application of apamin (500 nM). **(i)** Recording protocol used and the typical current traces recorded. **(ii)** Time course for current amplitudes measured in **(i)**. **(iii)** Summarized data for experiments shown in **(i,ii)** (paired *t*-test, ****p < 0.0001, *^n.s.^p* > 0.05, *n* = 7). **(E)** SK channel blocker apamin (500 nM) did not induce spontaneous firing of the DRN 5-HT neurons. **(i)** Representative traces of action potential spikes recorded using “loose patch” under current-clamp configuration. **(ii)** Example time course of the spontaneous firing frequency in a 5-HT neuron, under the influence of apamin and PE. **(iii)** Summarized data for the effects of apamin and PE on the spontaneous firing frequency (Wilcoxon matched-pairs signed-rank test, ***p < 0.001, *n* = 12).

Ca^2+^-activated K^+^ (SK) channels have been shown to regulate the firing pattern of central neurons, including DRN 5-HT neurons (Pan et al., 1994; Wagner et al., 2001; Adelman et al., 2012; Gocho et al., 2013; Matschke et al., 2018). However, conflicting results have been reported regarding the role of SK channels in PE-induced firing of DRN 5-HT neurons (Pan et al., 1994; Maingret et al., 2008). Moreover, the molecular identities of the SK currents in DRN 5-HT neurons are not clear. Three isoforms of SK channels (SK1, SK2, and SK3) have been described (Adelman et al., 2012). We first examined the expression profiles of these SK channels in DRN 5-HT neurons using single-cell PCR (Figure 4A) and immunofluorescence approaches (Figure 4B). We observed strong expression of SK2 and SK3 channels in DRN 5-HT neurons at both mRNA and protein levels, suggesting that SK currents in DRN 5-HT neurons are mediated by these SK channels. It has been suggested that SK currents are primarily involved in slow afterhyperpolarization (sAHP) during action potential firing (Pan et al., 1994; Wagner et al., 2001; Adelman et al., 2012; Gocho et al., 2013; Matschke et al., 2018). We isolated the AHP outward currents (I_AHP_) encoded by SK channels using a one-step voltage-clamp protocol (Matschke et al., 2018), the tail currents measured at the beginning of −60 mV following a depolarizing potential of 0 mV (Figure 4Ci). PE (10 μM) significantly inhibited the I_AHP_ in DRN 5-HT neurons, from initial current amplitudes of 44.04 ± 6.64 to 25.18 ± 6.28 pA (*n* = 6, *p* < 0.01, paired *t*-test, Figure 4C). It appears that PE only partially inhibited SK currents because apamin, a selective SK channel blocker, had a stronger inhibition on SK currents when applied either after (Figures 4Ci,ii,iii) or before (Figures 4Di,ii,iii) of PE. However, even with maximal inhibition of SK currents, apamin did not induce significant, sustained spontaneous firing of DRN 5-HT neurons (only a transient increase was occasionally observed, e.g., Figure 4Eii), although subsequent application of PE elicited firing of these neurons (Figure 4E). These results suggest that inhibition of SK channels does not trigger spontaneous firing of DRN 5-HT neurons.

### Multiple K^+^ Conductances Are Involved in the Phenylephrine-Induced Spontaneous Firing of the Dorsal Raphe Nucleus 5-HT Neurons

**FIGURE 5:**
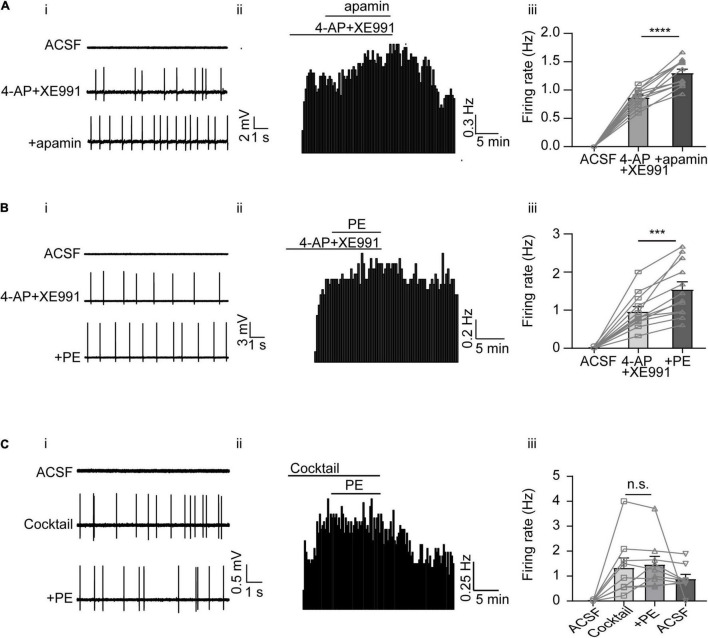
The overall contribution of A-type, M-type, and SK channels to the PE-induced spontaneous firing of the DRN 5-HT neurons. **(A)** SK channel blocker apamin further increased the induced spontaneous firing of the DRN 5-HT neurons, following the application of 4AP and XE991. **(i)** Representative traces of action potential spikes recorded using “loose patch” under current-clamp configuration performed on a DRN 5-HT neuron; the effects of 4AP + XE991 and apamin are shown. **(ii)** Example time course of the spontaneous firing frequency in a 5-HT neuron, under the influence of 4AP + XE991 and apamin. **(iii)** Summarized effects of 4AP + XE991 and apamin on the spontaneous firing frequency (paired *t*-test, ****p < 0.0001, *n* = 12). **(B)** PE further increased the induced spontaneous firing of 5-HT neurons in the DRN, following the application of 4AP and XE991. **(i)** Representative traces of action potential spikes recorded using “loose patch” under current-clamp configuration performed on a DRN 5-HT neuron; the effects of 4AP + XE991 and PE are shown. **(ii)** Example time course of the spontaneous firing frequency in a 5-HT neuron, under the influence of 4AP + XE991 and PE. **(iii)** Summarized effects of 4AP + XE991 and PE on the spontaneous firing frequency (paired *t*-test, ***p < 0.001, *n* = 12). **(C)** Three K^+^ channel blockers, 4AP, XE991, and apamin, together totally excluded further effect of PE on the spontaneous firing of the DRN 6-HT neurons. **(i)** Representative traces of action potential spikes recorded using “loose patch” under current-clamp configuration performed on a DRN 5-HT neuron; the effects of cocktail blockers (4-AP, XE991, and apamin) and PE are shown. **(ii)** Example time course of the spontaneous firing frequency in a 5-HT neuron, under the influence of cocktail blockers and PE. **(iii)** Summarized effects of cocktail blockers and PE on the spontaneous firing frequency (paired *t*-test, *^n.s.^p* > 0.05, *n* = 9).

While blocking SK channels with apamin did not elicit as strong firing activity as blocking A-type and KCNQ/M channels, apamin triggered transient, sparse firing activity in some DRN 5-HT neurons (see, e.g., Figure 4Eii). This prompted us to further test the effect of apamin. We first induced firing of DRN 5-HT neurons using both XE991 and 4-AP to block A-type and KCNQ/M K^+^ currents, and then additionally applied apamin. As shown in Figure 5A, the firing rate was further increased after apamin addition (from 0.87 ± 0.05 to 1.30 ± 0.07 Hz, *n* = 12, *p* < 0.0001, paired *t*-test, Figure 5A). Interestingly, PE also induced further firing activity when administered in addition to XE991 and 4-AP (from 0.96 ± 0.13 to 1.54 ± 0.20 Hz, *n* = 12, *p* < 0.001, paired *t*-test, Figure 5B), likely due to inhibition of SK channels. Taken together, these results imply that inhibition of SK currents, although not directly triggering spontaneous firing of DRN 5-HT neurons, contributed to the PE-induced firing activity of DRN 5-HT neurons.

Finally, to prove that the K^+^ conductance of A-type, KCNQ/M, and SK channels are sufficient components for the PE-induced spontaneous firing of DRN 5-HT neurons, a cocktail of the blockers for these K^+^ channels (4-AP, XE991, and apamin) was tested. The blocker cocktail evoked spontaneous firing of DRN 5-HT neurons (1.34 ± 0.39 Hz, *n* = 9, Figure 5C), which was not further enhanced by subsequent addition of PE (1.47 ± 0.32 Hz, *n* = 9, *p* > 0.05, paired *t*-test). These results suggest that blocking K^+^ channels (A-type, KCNQ/M, and SK currents) is a sufficient mechanism to trigger PE-induced spontaneous firing of DRN 5-HT neurons.

## Discussion

In this study, we investigated the mechanism for PE-induced spontaneous firing activity in DRN 5-HT neurons. The results show that inhibition of K^+^ currents from three K^+^ channel families, A-type, KCNQ/M, and SK channels, likely underlies PE-induced firing of DRN 5-HT neurons.

Phenylephrine induced spontaneous firing of DRN 5-HT neurons through α1-adrenoceptor because this excitatory effect was blocked by prazosin (a specific α1-adrenoceptor antagonist). Furthermore, this excitatory effect was maintained in the presence of a cocktail of ionotropic receptor blockers that inhibit NMDA receptors AMPA/kainite receptors, GABA_A_ receptors, and glycine receptors, suggesting that PE directly activates α1-adrenoceptor on DRN 5-HT neurons.

Although PE inhibition of A-type currents (I_A_) through α1-adrenoceptor in DRN 5-HT neurons was described long ago (Aghajanian, 1985), the contribution of this modulation to the firing activity of DRN 5-HT neurons has not been established. Moreover, the identity of the I_A_-correlated subtype channels in these neurons is unknown. In this regard, the results presented in this study provide a clear conclusion that PE-induced inhibition of I_A_, carried by the Kv4.2 and 4.3 channel subfamily, contributes to the PE-induced spontaneous firing of DRN 5-HT neurons.

Several studies have shown a marked control of neuronal excitability by A-type currents (IA) (Carrasquillo et al., 2012; Zhao et al., 2016; Yu et al., 2019). At least six Kv channels, as pore-forming α subunits, can rapidly generate activating and inactivating K^+^ currents with properties similar to neuronal I_A_, including Kv1.4 (KCNA4), Kv3.3 (KCNC3), Kv3.4 (KCNC4), Kv4.1 (KCND1), Kv4.2 (KCND2), and Kv4.3 (KCND3) (Stuhmer et al., 1989; Baldwin et al., 1991; Pak et al., 1991; Vega-Saenz de Miera et al., 1992; Ritter et al., 2012). I_A_ encoded by these different Kv α subunits show unique properties, whereas I_A_ encoded by Kv1.4 and Kv4s is activated at low voltage, I_A_ encoded by Kv3.3 and 3.4 are activated at high-voltage (>−20 mV) (Vega-Saenz de Miera et al., 1992). Our results are partially in agreement with previous evidence that the Kv4.3 transcript is abundant in the rat raphe, whereas Kv4.1 and 4.2 signals are negligible (Serodio and Rudy, 1998). Our results suggest that both Kv4.2 and 4.3 are highly expressed in mouse DRN 5-HT neurons, whereas Kv1.4 and Kv4.1 are negligible. Thus, Kv4.2 and 4.3 channels are most likely the molecular correlates of sub-threshold A-type currents in 5-HT neurons, although Kv4.2 could play a more dominant role given the different properties of these two Kv4 channels in action potential firing (Carrasquillo et al., 2012).

However, the I_A_ blocker 4-AP did not induce spontaneous firing of DRN 5-HT neurons as efficiently as PE, even at a maximal concentration (4 mM), suggesting that mechanisms other than I_A_ inhibition are involved in the PE-induced spontaneous firing activity of 5-HT neurons. Inhibition of KCNQ/M currents (I_M_) and low-conductance Ca^2+^-activated K^+^ (SK) currents (I_*SK*_) are the main candidates for this. These two-channel families are widely expressed in the central nervous system and play a key role in the intrinsic excitability of neurons (Wang et al., 1998; Stocker and Pedarzani, 2000; Sailer et al., 2004; Adelman et al., 2012), and more importantly, they have a high propensity for Gq-coupled (like α1-Ars) neuromodulation (Marrion et al., 1989; Bernheim et al., 1992; Maingret et al., 2008; Adelman et al., 2012; Kuo et al., 2016).

Much evidence suggests that modulation of I_M_ has profound effects on neuronal excitability (Brown and Passmore, 2009; Zhao et al., 2017; Su et al., 2019), and the KCNQ/M channel is a target of modulation by Gq-coupled receptors, including α1-Ars (Suh et al., 2004; Delmas and Brown, 2005; Kuo et al., 2016). We have shown in this study that pharmacological inhibition of I_M_ by the specific blocker XE911 also induced spontaneous firing of DRN 5-HT neurons and that PE at the concentration that triggers spontaneous firing inhibited I_M_ in DRN 5-HT neurons. These results demonstrate that I_M_ is another mechanism for the PE-induced spontaneous firing in DRN 5-HT neurons. Since KCNQ2, Q3, and Q4 are abundantly expressed in DRN 5-HT neurons, and all of these KCNQ subfamily members are known to produce I_M_ (Brown and Passmore, 2009), the PE-induced inhibition of I_M_ should originate from these KCNQ channels, contributing to the initiation of spontaneous firing.

As discussed above for I_A_ and I_M_, it could be similarly concluded that SK currents are also involved in the PE-induced spontaneous firing of DRN 5-HT neurons. However, the currents mediated by the SK channels are not involved in the initiation of the action potential like I_A_ and I_M_, but are mainly thought to contribute to the hyperpolarization following action potential and therefore regulate firing frequency (Adelman et al., 2012). This is probably due to the fact that they are usually not active during resting membrane potential and require elevated cytosolic Ca^2+^ levels to become active. This is consistent with our findings that inhibition of SK currents *per se* did not trigger significant firing activity, but rather increased firing frequency once firing was initiated by, for example, inhibition of I_A_ and I_M_. Our results suggest that of the three members of the SK channel family (SK1–SK3), SK2 and SK3 are the predominant types in DRN 5-HT neurons, consistent with previous findings (Stocker and Pedarzani, 2000; Sailer et al., 2004). Expression of mouse SK2 and SK3 was reported to produce functional, homomeric channels (Kohler et al., 1996; Shah and Haylett, 2000), whereas mouse SK1 cDNA did not produce functional plasma membrane channels (Benton et al., 2003). It should be noted that activation, rather than inhibition, of an apamin-sensitive late-AHP current by activation of α1-adrenoceptor in rat DRN 5-HT neurons has been reported (Pan et al., 1994), an observation that differs from our results. The different species used in this and our study may be one explanation, but other unknown mechanisms could also play a role.

Finally, the fact that inhibition of K^+^ conductance of the three channels discussed above completely excluded PE from further modulation of the firing activity clearly allows the conclusion that inhibition of K^+^ conductance is a mechanism sufficient to trigger PE-induced spontaneous firing of DRN 5-HT neurons.

In summary, our results suggest that A-type, KCNQ/M, and SK channels are the K^+^ channels that trigger PE-induced spontaneous firing in DRN 5-HT neurons. This mechanism is probably responsible for the neuronal modulation of DRN 5-HT neurons by the transmitter NE released from the terminals of the nerve fibers projecting from different brain regions. Whether this type of modulation is a unique mechanism for the DRN 5-HT neurons or a common mechanism for all central adrenergic neurons requires further investigation. Clarification of this question will help to understand the cellular mechanism of neuronal modulation and identify potential drug targets for therapeutic trials.

## Data Availability Statement

The original contributions presented in the study are included in the article/supplementary material, further inquiries can be directed to the corresponding author.

## Ethics Statement

The animal study was reviewed and approved by Laboratory Animal Ethical and Welfare Committee, Hebei Medical University.

## Author Contributions

HZ conceived, designed, and supervised the experiments. JW performed the experiments, acquired and analyzed the data, and prepared the figures. YW performed immunofluorescence of the brain slices and performed the some preliminary electrophysiological experiments. HZ and XD prepared the final version of the manuscript. All authors contributed to the article and approved the submitted version.

## Conflict of Interest

The authors declare that the research was conducted in the absence of any commercial or financial relationships that could be construed as a potential conflict of interest.

## Publisher’s Note

All claims expressed in this article are solely those of the authors and do not necessarily represent those of their affiliated organizations, or those of the publisher, the editors and the reviewers. Any product that may be evaluated in this article, or claim that may be made by its manufacturer, is not guaranteed or endorsed by the publisher.

## Abbreviations

ACSF: artificial cerebrospinal fluid
DRN: dorsal raphe nucleus
HEPES: *N*-2-hydroxyethylpiperazine-*N*′-2-ethanesulfonic acid
I_A_: A-type currents
I_M_: M-type current
LC: locus coeruleus
MDD: major depressive disorder
NE: norepinephrine
PE: phenylephrine
PFA: paraformaldehyde
RTN: retrotrapezoid nucleus
SK: calcium-activated small-conductance K^+^ channels
TEA: tetraethylammonium
TPH: tryptophan hydroxylase
4-AP: 4-aminopyridine.

## References

[B1] Adelman J. P. Maylie J. Sah P. (2012). Small-conductance Ca2+-activated K+ channels: form and function. *Annu. Rev. Physiol.* 74 245–269. 10.1146/annurev-physiol-020911-153336 21942705

[B2] Aghajanian G. K. (1985). Modulation of a transient outward current in serotonergic neurones by alpha 1-adrenoceptors. *Nature* 315 501–503. 10.1038/315501a0 2582271

[B3] Aghajanian G. K. Foote W. E. Sheard M. H. (1968). Lysergic acid diethylamide: sensitive neuronal units in the midbrain raphe. *Science* 161 706–708. 10.1126/science.161.3842.706 4874578

[B4] Aghajanian G. K. Vandermaelen C. P. (1982). Intracellular recordings from serotonergic dorsal raphe neurons: pacemaker potentials and the effect of LSD. *Brain Res.* 238 463–469. 10.1016/0006-8993(82)90124-x 6284300

[B5] Allers K. A. Sharp T. (2003). Neurochemical and anatomical identification of fast- and slow-firing neurones in the rat dorsal raphe nucleus using juxtacellular labelling methods in vivo. *Neuroscience* 122 193–204. 10.1016/s0306-4522(03)00518-9 14596860

[B6] Baldwin T. J. Tsaur M. L. Lopez G. A. Jan Y. N. Jan L. Y. (1991). Characterization of a mammalian cDNA for an inactivating voltage-sensitive K+ channel. *Neuron* 7 471–483. 10.1016/0896-6273(91)90299-f 1840649

[B7] Benton D. C. Monaghan A. S. Hosseini R. Bahia P. K. Haylett D. G. Moss G. W. (2003). Small conductance Ca2+-activated K+ channels formed by the expression of rat SK1 and SK2 genes in HEK 293 cells. *J. Physiol.* 553 13–19. 10.1113/jphysiol.2003.054551 14555714 PMC2343499

[B8] Bernheim L. Mathie A. Hille B. (1992). Characterization of muscarinic receptor subtypes inhibiting Ca2+ current and M current in rat sympathetic neurons. *Proc. Natl. Acad. Sci. U. S. A.* 89 9544–9548. 10.1073/pnas.89.20.9544 1329101 PMC50168

[B9] Bromberg-Martin E. S. Hikosaka O. Nakamura K. (2010). Coding of task reward value in the dorsal raphe nucleus. *J. Neurosci.* 30 6262–6272. 10.1523/JNEUROSCI.0015-10.2010 20445052 PMC3467971

[B10] Brown D. A. Passmore G. M. (2009). Neural KCNQ (Kv7) channels. *Br. J. Pharmacol.* 156 1185–1195. 10.1111/j.1476-5381.2009.00111.x 19298256 PMC2697739

[B11] Burlet S. Tyler C. J. Leonard C. S. (2002). Direct and indirect excitation of laterodorsal tegmental neurons by Hypocretin/Orexin peptides: implications for wakefulness and narcolepsy. *J. Neurosci.* 22 2862–2872. 10.1523/JNEUROSCI.22-07-02862.2002 11923451 PMC6758338

[B12] Carrasquillo Y. Burkhalter A. Nerbonne J. M. (2012). A-type K+ channels encoded by Kv4.2, Kv4.3 and Kv1.4 differentially regulate intrinsic excitability of cortical pyramidal neurons. *J. Physiol.* 590 3877–3890. 10.1113/jphysiol.2012.229013 22615428 PMC3476638

[B13] Commons K. G. (2020). Dorsal raphe organization. *J. Chem. Neuroanat.* 110:101868. 10.1016/j.jchemneu.2020.101868 33031916 PMC8530532

[B14] Delmas P. Brown D. A. (2005). Pathways modulating neural KCNQ/M (Kv7) potassium channels. *Nat. Rev. Neurosci.* 6 850–862. 10.1038/nrn1785 16261179

[B15] Gocho Y. Sakai A. Yanagawa Y. Suzuki H. Saitow F. (2013). Electrophysiological and pharmacological properties of GABAergic cells in the dorsal raphe nucleus. *J. Physiol. Sci.* 63 147–154. 10.1007/s12576-012-0250-7 23275149 PMC3579464

[B16] Hayashi K. Nakao K. Nakamura K. (2015). Appetitive and aversive information coding in the primate dorsal raphe nucleus. *J. Neurosci.* 35 6195–6208. 10.1523/JNEUROSCI.2860-14.2015 25878290 PMC6605165

[B17] Hu F. Zhou J. Lu Y. Guan L. Wei N. N. Tang Y. Q. (2019). Inhibition of Hsp70 Suppresses Neuronal Hyperexcitability and Attenuates Epilepsy by Enhancing A-Type Potassium Current. *Cell Rep.* 26 .168–181. 30605673 10.1016/j.celrep.2018.12.032

[B18] Itri J. N. Vosko A. M. Schroeder A. Dragich J. M. Michel S. Colwell C. S. (2010). Circadian regulation of a-type potassium currents in the suprachiasmatic nucleus. *J. Neurophysiol.* 103 632–640. 19939959 10.1152/jn.00670.2009 PMC2822681

[B19] Judge S. J. Gartside S. E. (2006). Firing of 5-HT neurones in the dorsal and median raphe nucleus in vitro shows differential alpha1-adrenoceptor and 5-HT1A receptor modulation. *Neurochem. Int.* 48 100–107. 10.1016/j.neuint.2005.09.003 16256247

[B20] Kawashima T. (2018). The role of the serotonergic system in motor control. *Neurosci. Res.* 129 32–39. 10.1016/j.neures.2017.07.005 28774814

[B21] Kohler M. Hirschberg B. Bond C. T. Kinzie J. M. Marrion N. V. Maylie J. (1996). Small-conductance, calcium-activated potassium channels from mammalian brain. *Science* 273 1709–1714. 10.1126/science.273.5282.1709 8781233

[B22] Kuo F. S. Falquetto B. Chen D. Oliveira L. M. Takakura A. C. Mulkey D. K. (2016). In vitro characterization of noradrenergic modulation of chemosensitive neurons in the retrotrapezoid nucleus. *J. Neurophysiol.* 116 1024–1035. 10.1152/jn.00022.2016 27306669 PMC5009215

[B23] Leonard B. E. (2002). Neuropsychopharmacology—The fifth generation of progress. Edited by K. L. Davis, D. Charney, J. T. Coyle, C. Nemeroff. Lippincott, Williams and Wilkins: Philadelphia, 2002. ISBN: 0-7817-2837-1. Price: $189. Pages: 2080. *Hum. Psychol.* 17 433–433.

[B24] Li Y. Dalphin N. Hyland B. I. (2013). Association with reward negatively modulates short latency phasic conditioned responses of dorsal raphe nucleus neurons in freely moving rats. *J. Neurosci.* 33 5065–5078. 10.1523/JNEUROSCI.5679-12.2013 23486976 PMC6618993

[B25] Lucki I. (1998). The spectrum of behaviors influenced by serotonin. *Biol. Psychiatry* 44 151–162. 10.1016/s0006-3223(98)00139-5 9693387

[B26] Maingret F. Coste B. Hao J. Giamarchi A. Allen D. Crest M. (2008). Neurotransmitter modulation of small-conductance Ca2+-activated K+ channels by regulation of Ca2+ gating. *Neuron* 59 439–449. 10.1016/j.neuron.2008.05.026 18701069 PMC2651825

[B27] Marrion N. V. Smart T. G. Marsh S. J. Brown D. A. (1989). Muscarinic suppression of the M-current in the rat sympathetic ganglion is mediated by receptors of the M1-subtype. *Br. J. Pharmacol.* 98 557–573. 10.1111/j.1476-5381.1989.tb12630.x 2819334 PMC1854721

[B28] Matschke L. A. Rinne S. Snutch T. P. Oertel W. H. Dolga A. M. Decher N. (2018). Calcium-activated SK potassium channels are key modulators of the pacemaker frequency in locus coeruleus neurons. *Mol. Cell Neurosci.* 88 330–341. 10.1016/j.mcn.2018.03.002 29524627

[B29] Matthews G. A. Nieh E. H. Vander Weele C. M. Halbert S. A. Pradhan R. V. Yosafat A. S. (2016). Dorsal Raphe Dopamine Neurons Represent the Experience of Social Isolation. *Cell* 164 617–631. 10.1016/j.cell.2015.12.040 26871628 PMC4752823

[B30] Monti J. M. (2010). The role of dorsal raphe nucleus serotonergic and non-serotonergic neurons, and of their receptors, in regulating waking and rapid eye movement (REM) sleep. *Sleep Med. Rev.* 14 319–327. 10.1016/j.smrv.2009.10.003 20153670

[B31] Nakamura K. Matsumoto M. Hikosaka O. (2008). Reward-dependent modulation of neuronal activity in the primate dorsal raphe nucleus. *J. Neurosci.* 28 5331–5343. 10.1523/JNEUROSCI.0021-08.2008 18480289 PMC3329731

[B32] Ohmura Y. Tsutsui-Kimura I. Sasamori H. Nebuka M. Nishitani N. Tanaka K. F. (2020). Different roles of distinct serotonergic pathways in anxiety-like behavior, antidepressant-like, and anti-impulsive effects. *Neuropharmacology* 167:107703. 10.1016/j.neuropharm.2019.107703 31299228

[B33] Pak M. D. Baker K. Covarrubias M. Butler A. Ratcliffe A. Salkoff L. (1991). mShal, a subfamily of A-type K+ channel cloned from mammalian brain. *Proc. Natl. Acad. Sci. U. S. A.* 88 4386–4390. 10.1073/pnas.88.10.4386 2034678 PMC51664

[B34] Pan Z. Z. Grudt T. J. Williams J. T. (1994). Alpha 1-adrenoceptors in rat dorsal raphe neurons: regulation of two potassium conductances. *J. Physiol.* 478 437–447. 10.1113/jphysiol.1994.sp020263 7525947 PMC1155664

[B35] Pan Z. Z. Williams J. T. Osborne P. B. (1990). Opioid actions on single nucleus raphe magnus neurons from rat and guinea-pig in vitro. *J. Physiol.* 427 519–532. 10.1113/jphysiol.1990.sp018185 1976803 PMC1189944

[B36] Pedarzani P. McCutcheon J. E. Rogge G. Jensen B. S. Christophersen P. Hougaard C. (2005). Specific enhancement of SK channel activity selectively potentiates the afterhyperpolarizing current I(AHP) and modulates the firing properties of hippocampal pyramidal neurons. *J. Biol. Chem.* 280 41404–41411. 10.1074/jbc.M509610200 16239218

[B37] Prakash N. Stark C. J. Keisler M. N. Luo L. Der-Avakian A. Dulcis D. (2020). Serotonergic Plasticity in the Dorsal Raphe Nucleus Characterizes Susceptibility and Resilience to Anhedonia. *J. Neurosci.* 40 569–584. 10.1523/JNEUROSCI.1802-19.2019 31792153 PMC6961996

[B38] Ritter D. M. Ho C. O’Leary M. E. Covarrubias M. (2012). Modulation of Kv3.4 channel N-type inactivation by protein kinase C shapes the action potential in dorsal root ganglion neurons. *J. Physiol.* 590 145–161. 10.1113/jphysiol.2011.218560 22063632 PMC3300053

[B39] Sailer C. A. Hu H. Kaufmann W. A. Trieb M. Schwarzer C. Storm J. F. (2002). Regional differences in distribution and functional expression of small-conductance Ca2+-activated K+ channels in rat brain. *J. Neurosci.* 22 9698–9707. 10.1523/JNEUROSCI.22-22-09698.2002 12427825 PMC6757844

[B40] Sailer C. A. Kaufmann W. A. Marksteiner J. Knaus H. G. (2004). Comparative immunohistochemical distribution of three small-conductance Ca2+-activated potassium channel subunits, SK1, SK2, and SK3 in mouse brain. *Mol. Cell Neurosci.* 26 458–469. 10.1016/j.mcn.2004.03.002 15234350

[B41] Schweimer J. V. Ungless M. A. (2010). Phasic responses in dorsal raphe serotonin neurons to noxious stimuli. *Neuroscience* 171 1209–1215. 10.1016/j.neuroscience.2010.09.058 20888395

[B42] Serodio P. Rudy B. (1998). Differential expression of Kv4 K+ channel subunits mediating subthreshold transient K+ (A-type) currents in rat brain. *J. Neurophysiol.* 79 1081–1091. 10.1152/jn.1998.79.2.1081 9463463

[B43] Serôdio P. Vega-Saenz de Miera E. Rudy B. (1996). Cloning of a novel component of A-type K+ channels operating at subthreshold potentials with unique expression in heart and brain. *J. Neurophysiol.* 75 2174–2179. 10.1152/jn.1996.75.5.2174 8734615

[B44] Shah M. Haylett D. G. (2000). The pharmacology of hSK1 Ca2+-activated K+ channels expressed in mammalian cell lines. *Br. J. Pharmacol.* 129 627–630. 10.1038/sj.bjp.0703111 10683185 PMC1571896

[B45] Soiza-Reilly M. Commons K. G. (2011). Glutamatergic drive of the dorsal raphe nucleus. *J. Chem. Neuroanat.* 41 247–255. 10.1016/j.jchemneu.2011.04.004 21550397 PMC3150565

[B46] Song W. J. Tkatch T. Baranauskas G. Ichinohe N. Kitai S. T. Surmeier D. J. (1998). Somatodendritic depolarization-activated potassium currents in rat neostriatal cholinergic interneurons are predominantly of the A type and attributable to coexpression of Kv4.2 and Kv4.1 subunits. *J. Neurosci.* 18 3124–3137. 10.1523/JNEUROSCI.18-09-03124.1998 9547221 PMC6792663

[B47] Stocker M. Pedarzani P. (2000). Differential distribution of three Ca(2+)-activated K(+) channel subunits, SK1, SK2, and SK3, in the adult rat central nervous system. *Mol. Cell Neurosci.* 15 476–493. 10.1006/mcne.2000.0842 10833304

[B48] Stuhmer W. Ruppersberg J. P. Schroter K. H. Sakmann B. Stocker M. Giese K. P. (1989). Molecular basis of functional diversity of voltage-gated potassium channels in mammalian brain. *EMBO J.* 8 3235–3244. 10.1002/j.1460-2075.1989.tb08483.x 2555158 PMC401447

[B49] Su M. Li L. Wang J. Sun H. Zhang L. Zhao C. (2019). Kv7.4 Channel Contribute to Projection-Specific Auto-Inhibition of Dopamine Neurons in the Ventral Tegmental Area. *Front. Cell Neurosci.* 13:557. 10.3389/fncel.2019.00557 31920557 PMC6930245

[B50] Suh B. C. Horowitz L. F. Hirdes W. Mackie K. Hille B. (2004). Regulation of KCNQ2/KCNQ3 current by G protein cycling: the kinetics of receptor-mediated signaling by Gq. *J. Gen. Physiol.* 123 663–683. 10.1085/jgp.200409029 15173220 PMC2234571

[B51] Vandermaelen C. P. Aghajanian G. K. (1983). Electrophysiological and pharmacological characterization of serotonergic dorsal raphe neurons recorded extracellularly and intracellularly in rat brain slices. *Brain Res.* 289 109–119. 10.1016/0006-8993(83)90011-2 6140982

[B52] Vega-Saenz de Miera E. Moreno H. Fruhling D. Kentros C. Rudy B. (1992). Cloning of ShIII (Shaw-like) cDNAs encoding a novel high-voltage-activating, TEA-sensitive, type-A K+ channel. *Proc. Biol. Sci.* 248 9–18. 10.1098/rspb.1992.0036 1381835

[B53] Wagner E. J. Ronnekleiv O. K. Kelly M. J. (2001). The noradrenergic inhibition of an apamin-sensitive, small-conductance Ca2+-activated K+ channel in hypothalamic gamma-aminobutyric acid neurons: pharmacology, estrogen sensitivity, and relevance to the control of the reproductive axis. *J. Pharmacol. Exp. Ther.* 299 21–30. 11561059

[B54] Wang H. S. Pan Z. Shi W. Brown B. S. Wymore R. S. Cohen I. S. (1998). KCNQ2 and KCNQ3 potassium channel subunits: molecular correlates of the M-channel. *Science* 282 1890–1893. 10.1126/science.282.5395.1890 9836639

[B55] Yao J. A. Tseng G. N. (1994). Modulation of 4-AP block of a mammalian A-type K channel clone by channel gating and membrane voltage. *Biophys. J.* 67 130–142. 10.1016/S0006-3495(94)80462-X 7918980 PMC1225342

[B56] Yu S. Zhang Y. Zhao X. Chang Z. Wei Y. Sun Y. (2019). Cholecystokinin type B receptor-mediated inhibition of A-type K(+) channels enhances sensory neuronal excitability through the phosphatidylinositol 3-kinase and c-Src-dependent JNK pathway. *Cell Commun. Signal.* 17:68. 10.1186/s12964-019-0385-8 31215470 PMC6582535

[B57] Zhao C. Su M. Wang Y. Li X. Zhang Y. Du X. (2017). Selective Modulation of K(+) Channel Kv7.4 Significantly Affects the Excitability of DRN 5-HT Neurons. *Front. Cell Neurosci.* 11:405. 10.3389/fncel.2017.00405 29311835 PMC5735115

[B58] Zhao X. Zhang Y. Qin W. Cao J. Zhang Y. Ni J. (2016). Serotonin type-1D receptor stimulation of A-type K(+) channel decreases membrane excitability through the protein kinase A- and B-Raf-dependent p38 MAPK pathways in mouse trigeminal ganglion neurons. *Cell Signal.* 28 979–988. 10.1016/j.cellsig.2016.05.004 27156838

[B59] Zou W. J. Song Y. L. Wu M. Y. Chen X. T. You Q. L. Yang Q. (2020). A discrete serotonergic circuit regulates vulnerability to social stress. *Nat. Commun.* 11:4218. 10.1038/s41467-020-18010-w 32839452 PMC7445164

